# Supplementary figures and images for: Quantitative SARS-CoV-2 subgenomic RNA as a surrogate marker for viral infectivity: Comparison between culture isolation and direct sgRNA quantification
Source: PLoS One. 2023 Sep 1;18(9):e0291120. doi: 10.1371/journal.pone.0291120 (PMC10473502; doi:10.1371/journal.pone.0291120)

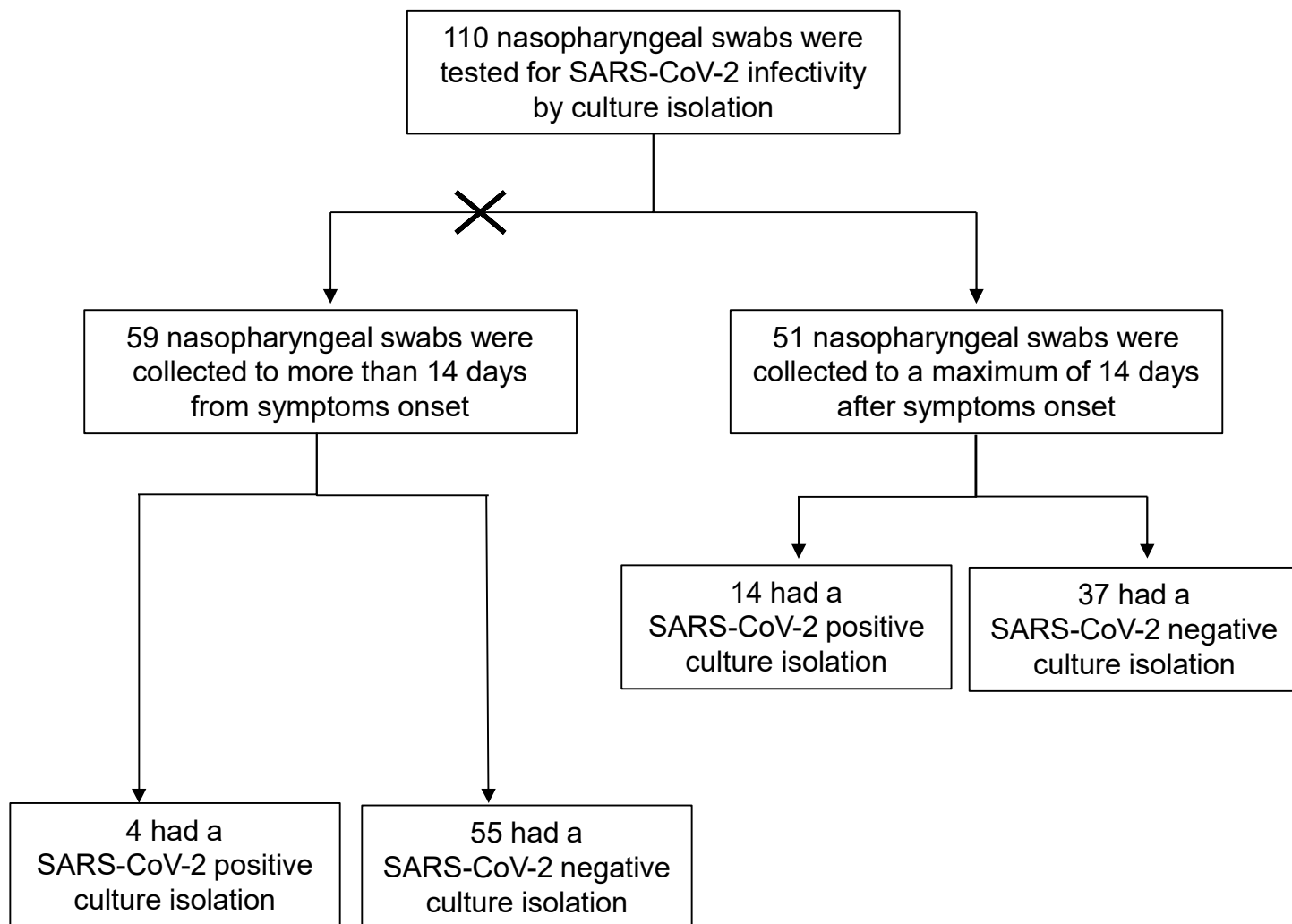

Supplementary Fig. 1. **Selection criteria for the 51 nasopharyngeal swabs included in the study.**

Supplement: S1 Fig — (PDF) [file pone.0291120.s001.pdf]
