# Supplementary material for: Quantitative SARS-CoV-2 subgenomic RNA as a surrogate marker for viral infectivity: Comparison between culture isolation and direct sgRNA quantification
Source: PLoS One. 2023 Sep 1;18(9):e0291120. doi: 10.1371/journal.pone.0291120 (PMC10473502; doi:10.1371/journal.pone.0291120)
Supplement: S2 Fig — In each panel, the first and second wells represent the negative and positive reaction control, respectively. (PDF) [file pone.0291120.s002.pdf]

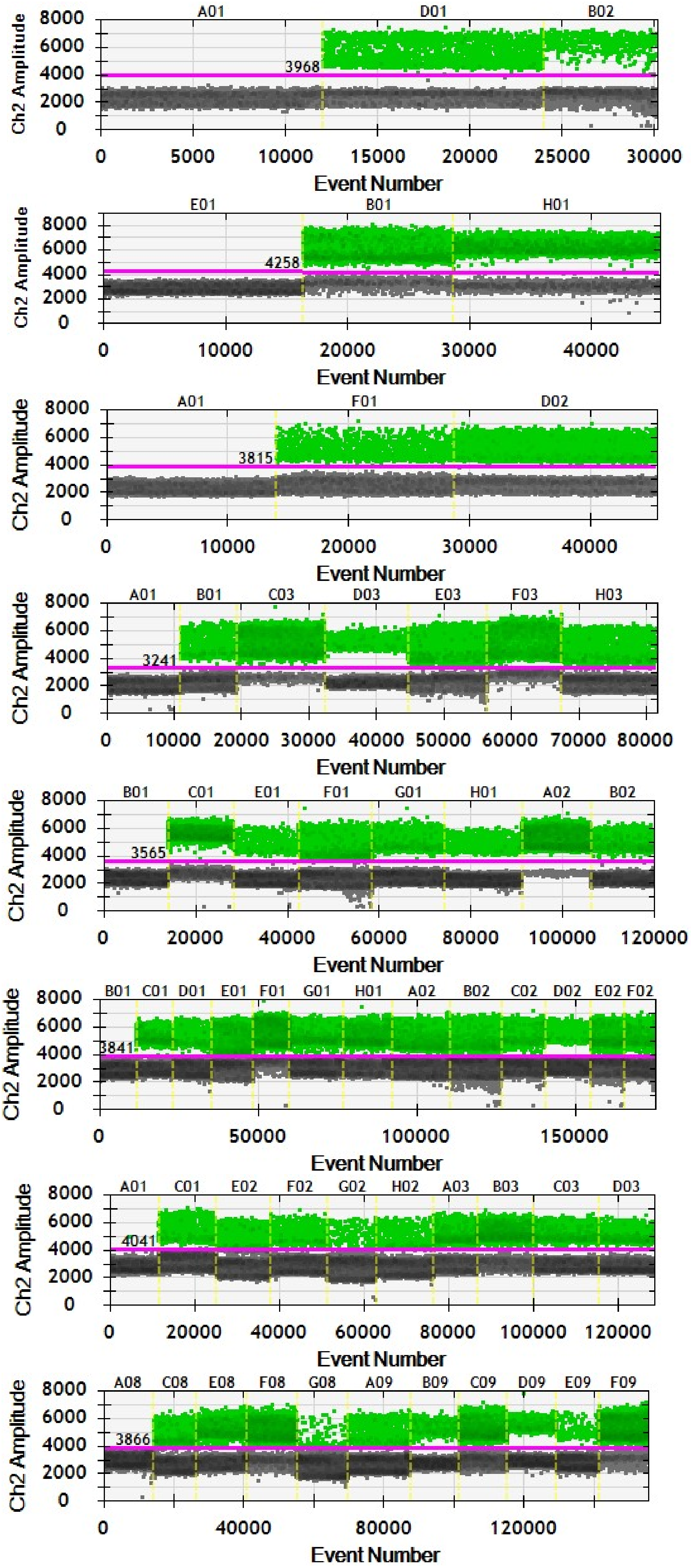
Ch2 Amplitude
Event Number
A01 D01 B02
3968
E01 B01 H01
4258
A01 F01 D02
3815
A01 B01 C03 D03 E03 F03 H03
3241
B01 C01 E01 F01 G01 H01 A02 B02
3565
B01 C01 D01 E01 F01 G01 H01 A02 B02 C02 D02 E02 F02
3841
A01 C01 E02 F02 G02 H02 A03 B03 C03 D03
4041
A08 C08 E08 F08 G08 A09 B09 C09 D09 E09 F09
3866

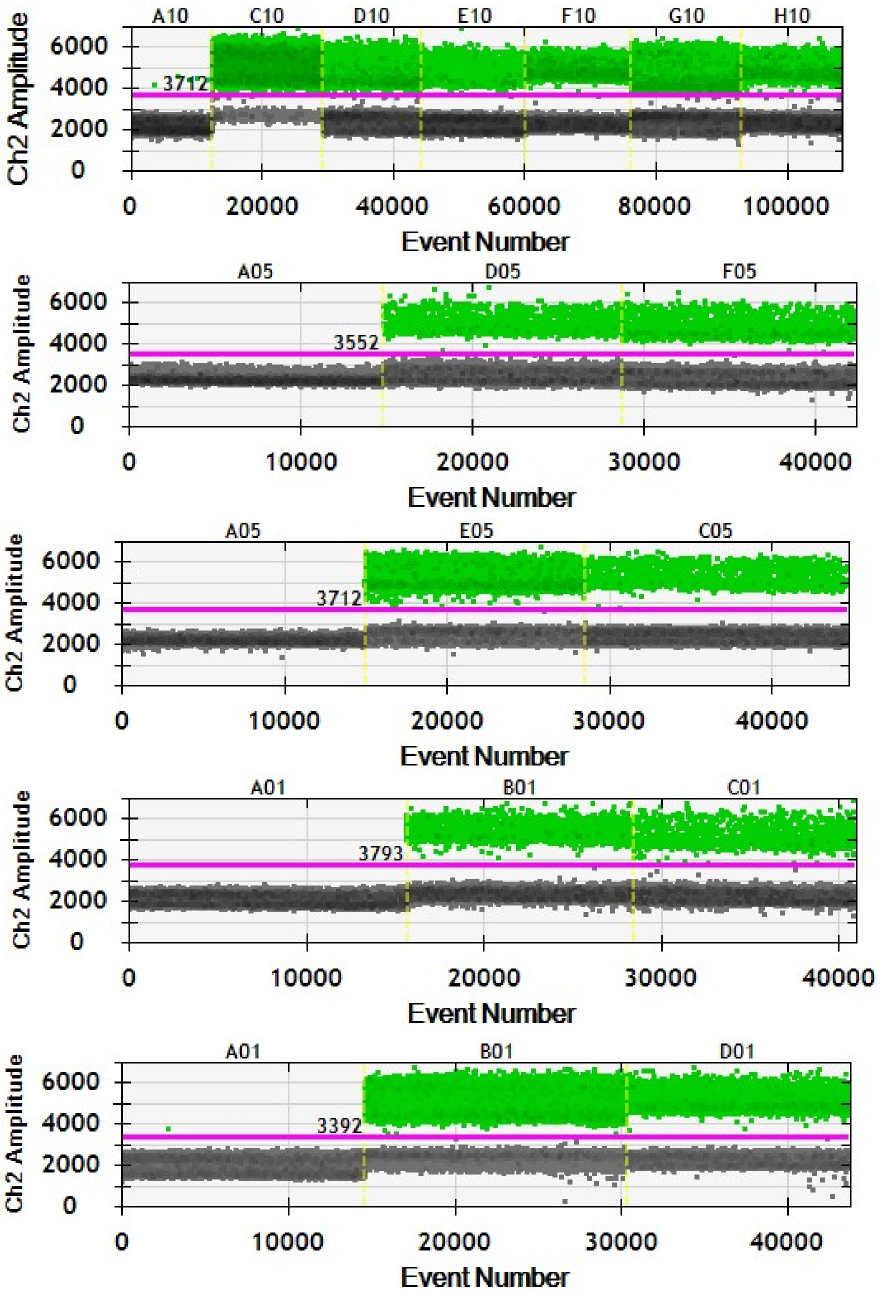

Supplementary Fig. 2. **Quantasoft panel for RNAse P housekeeping gene of the 51 nasopharyngeal swabs (one well per patient).** In each panel, the first and second wells represent the negative and positive reaction control, respectively.
