# Supplementary material for: Quantitative SARS-CoV-2 subgenomic RNA as a surrogate marker for viral infectivity: Comparison between culture isolation and direct sgRNA quantification
Source: PLoS One. 2023 Sep 1;18(9):e0291120. doi: 10.1371/journal.pone.0291120 (PMC10473502; doi:10.1371/journal.pone.0291120)
Supplement: S1 Table — (DOCX) [file pone.0291120.s003.docx]

Supplementary Table 1. **Primers and Probes used to quantify SARS-CoV-2 load and Subgenomic RNA.**

| RdRp Assay 1 | Forward | 5’- GACTTTGTGAATGAGTTTTACGC-3’ |
| --- | --- | --- |
|  | Reverse | 5’- AGCCACTAGACCTTGAGATGC-3’ |
|  | Probe | 5’- CACACAACAGCATCGTCAGA-3’ |
| RdRp Assay 2 | Forward | 5’-ATGAGCTTAGTCCTGTTG-3’ |
|  | Reverse | 5’-CTCCCTTTGTTGTGTTGT-3’ |
|  | Probe | 5’-AGATGTCTTGTGCTGCCGGTA-3’ |
| RdRp Assay 3 | Forward | 5’-GGTAACTGGTATGATTTCG-3’ |
|  | Reverse | 5’-CTGGTCAAGGTTAATATAGG-3’ |
|  | Probe | 5’-TCATACAAACCACGCCAGG-3’ |
| sgRNA N | Forward | 5’-CCAACCAACTTTCGATCTCTT-3’ |
|  | Reverse | 5’-GTGAACCAAGACGCAGTATTAT-3’ |
|  | Probe | 5’-TGGAGAACGCAGTGGGGCGCG-3’ |
| sgRNA E | Forward | 5’-CGATCTCTTGTAGATCTGTTCTC-3’ |
|  | Reverse | 5’-ATATTGCAGCAGTACGCACACA-3’ |
|  | Probe | 5’-ACACTAGCCATCCTTACTGCGCTTCG-3’ |
| RNAse P | Forward | 5’-AGATTTGGACCTGCGAGCG-3’ |
|  | Reverse | 5’-GAGCGGCTGTCTCCACAAGT-3’ |
|  | Probe | 5’-TTCTGACCTGAAGGCTCTGCGCG-3’ |

RdRp: RNA-dependent RNA-polymerase; sgRNA E: Envelope subgenomic RNA, sgRNA N: Nucleocapsid subgenomic RNA. RdRp Assays 2 and 3 referred to Institut Pasteur, Paris Protocol: Real-Time RT-PCR Assays for the Detection of SARS-CoV-2; Available online: https://www.who.int/docs/default-source/coronaviruse/real-time-rt-pcr-assays-for-the-detection-of-sars-cov-2-institut-pasteur-paris.pdf?sfvrsn=3662fcb6_2. sgRNA N Assay considered the forward primer already used by Telwatte S et al., “Novel RT-ddPCR assays for measuring the levels of subgenomic and genomic SARS-CoV-2 transcripts”. doi: 10.1016/j.ymeth.2021.04.011. sgRNA E Assay was adapted by Wölfel R. et al., “Virological assessment of hospitalized patients with COVID-2019.” doi: 10.1038/s41586-020-2196-x. RNAse P Assay referred to https://www.cdc.gov/coronavirus/2019-ncov/lab/multiplex.html?CDC_AA_refVal=https%3A%2F%2Fwww.cdc.gov%2Fcoronavirus%2F2019-ncov%2Flab%2Frt-pcr-panel-primer-probes.html
