# Supplementary material for: Quantitative SARS-CoV-2 subgenomic RNA as a surrogate marker for viral infectivity: Comparison between culture isolation and direct sgRNA quantification
Source: PLoS One. 2023 Sep 1;18(9):e0291120. doi: 10.1371/journal.pone.0291120 (PMC10473502; doi:10.1371/journal.pone.0291120)
Supplement: S2 Table — (DOCX) [file pone.0291120.s004.docx]

**Supplementary Table 2. SARS-CoV-2 genomic and subgenomic RNA load against culture isolation**

|  | **Overall** | **Culture isolation** | |  |
| --- | --- | --- | --- | --- |
|  |  | **SARS-CoV-2 Positive by culture** | **SARS-CoV-2 Negative by culture** | **P-value^a^** |
| **Patients, N** | 51 | 14 | 37 |  |
| **SARS-CoV-2 Viral Load:** | | | | |
| *Detectable* | 37 | 14 | 23 | **0.005** |
| *Not detectable* | 14 | 0 | 14 |  |
| *SARS-CoV-2 Viral Load (copies/mL)^b^* | 5,899 (705 – 105,817) | 3,174,612 (46,650 –10,000,000) | 1,913 (411 – 8,680) | **<0.001** |
| **Subgenomic N:** | | | | |
| *Detectable* | 19 | 14 | 5 | **<0.001** |
| *Not detectable* | 32 | 0 | 32 |  |
| *Subgenomic N*  *(copies/mL)* *^b^* | 1,680 (325 – 254,240) | 34,069 (325 – 306,600) | 770 (630 – 980) | 0.165 |
| **Subgenomic E:** | | | | |
| *Detectable* | 20 | 14 | 6 | **<0.001** |
| *Not detectable* | 31 | 0 | 31 |  |
| *Subgenomic E*  *(copies/mL)* *^b^* | 1,295 (238 – 906,850) | 140,700 (350 – 1,071,000) | 406 (210 – 770) | 0.083 |

Data are expressed as median (interquartile range, IQR), or N (%). ^a^ Fisher exact test and Mann-Whitney test were used for categorical and continuous variables, respectively. ^b^Among patients with detectable viral load. Statistically significant p-values are in bold.
