# Supplementary material for: Quantitative SARS-CoV-2 subgenomic RNA as a surrogate marker for viral infectivity: Comparison between culture isolation and direct sgRNA quantification
Source: PLoS One. 2023 Sep 1;18(9):e0291120. doi: 10.1371/journal.pone.0291120 (PMC10473502; doi:10.1371/journal.pone.0291120)
Supplement: S3 Table — (DOCX) [file pone.0291120.s005.docx]

Supplementary Table 3. **Demographic and clinical characteristics of patients against SARS-CoV-2 culture isolation vs subgenomic RNA concordance**

|  | Overall | SARS-CoV-2 culture isolation vs SARS-CoV-2 sgRNA | | |
| --- | --- | --- | --- | --- |
|  |  | Concordant -/- | Discordant -/+ | Concordant +/+ |
| **Patients, N** | 51 | 31 | 6 | 14 |
| **Males** | 25 (49.0) | 12 (38.7) | 4 (66.7) | 9 (64.3) |
| **Age (years)** | 59 (41-69) | 59 (41 – 72) | 42 (34 – 66) | 60 (48 – 67) |
| **Days from symptoms onset** | 8 (1-11) | 8 (1-11) | 11 (11-14) | 2 (1-7) |
| **COVID-19 manifestation at first positive nasopharyngeal swab^a^** | | | | |
| *Asymptomatic* | 7 (41.2) | 6 (46.2) | 0 (0.0) | 1 (25.0) |
| *Mild* | 5 (29.4) | 4 (30.7) | 0 (0.0) | 1 (25.0) |
| *Moderate/severe* | 5 (29.4) | 3 (23.1) | 0 (0.0) | 2 (50.0) |
| **Symptoms at first nasopharyngeal swab^a^** | | | | |
| *Fever* | 4 (23.5) | 4 (30.7) | 0 (0.0) | 0 (0.0) |
| *Cough* | 1 (5.9) | 0 (0.0) | 0 (0.0) | 1 (25.0) |
| *Dyspnoea* | 2 (11.8) | 2 (15.4) | 0 (0.0) | 0 (0.0) |
| *Evidence of interstitial pneumonia* | 5 (29.4) | 3 (23.1) | 0 (0.0) | 2 (50.0) |
| **Immuno-compromised patient^a^** | 6 (35.3) | 3 (23.1) | 0 (0.0) | 3 (75.0) |

Data are expressed as median (interquartile range, IQR), or N (%). COVID-19, Coronavirus Disease 2019. Negative concordant: negative culture isolation and negative sgRNA detection; Positive concordant: positive culture isolation and positive sgRNA detection; Discordant: negative culture isolation and positive sgRNA detection. SgRNA was considered positive (+) when at least one subgenomic RNA (N or E) was detectable

^a^Available for 17 patients.
