# Supplementary material for: Quantitative SARS-CoV-2 subgenomic RNA as a surrogate marker for viral infectivity: Comparison between culture isolation and direct sgRNA quantification
Source: PLoS One. 2023 Sep 1;18(9):e0291120. doi: 10.1371/journal.pone.0291120 (PMC10473502; doi:10.1371/journal.pone.0291120)
Supplement: S1 File — (DOCX) [file pone.0291120.s006.docx]

**S1 File: Full protocol used for the detection and quantification of SARS-CoV-2 subgenomic RNAs and viral load described in the manuscript: Quantitative SARS-CoV-2 subgenomic RNA as a surrogate marker for viral infectivity: comparison between culture isolation and direct sgRNA quantification.**

**MATERIAL AND METHODS**

### *SARS-CoV-2 load and Subgenomic RNA quantification by ddPCR*

Genomic RNA and sgRNA of SARS-CoV-2 were quantified using the QX200™ Droplet Digital™ PCR system (ddPCR, Bio-Rad Laboratories, Inc.). Genomic RNA was quantified using assays targeting the rdrp region, while subgenomic RNAs were quantified using assays targeting envelope and nucleocapsid transcripts. In addition, the assay targeting the housekeeping gene RNAseP was used as a reference [2].

More details about the assays and reagents used and the entire ddPCR workflow are given below.

**Before Starting**

- Wear clean gloves
- Clean pipettes and stand with 75% alcohol
- Thaw RNA samples and all reagents (One-Step RT-ddPCR Advanced Kit for Probes primers and probes, stock of fresh nuclease free water)
- Gently mix the RNA samples and mix thoroughly the reagents on the vortex
- Centrifuge the RNA samples and reagents with a spin
- Keep reagents on ice
- Required equipment (see Equipment section)
- Required consumables (see Consumables section)

**MATERIALS**

**Equipment**

- QX200™ Droplet Generator (Bio-Rad, Catalog #:1864002)

# Bio-Rad QX200 Droplet Reader (Bio-Rad, Catalog #:1814003)

# Bio-Rad PX1 PCR Plate Sealer (Bio-Rad, Catalog #:1814000)

# Bio-Rad C1000 Touch Thermal Cycler

# Gilson Single-Channel Pipettes P200L, P100L, P20L, P10L

# Gilson Multi-Channel Pipettes P8X200L

# Benchtop vortex

# 96-well plate cold block for AutoDG droplet generator

# 96-well plate cold block for ddPCR plates

**Reagents**

- One-Step RT-ddPCR Advanced Kit for Probes (Bio-Rad, Catalog #:1864022)
- Automated Droplet Generation Oil (Bio-Rad, Catalog #: 1863005)
- DNA/RNA Nuclease Free Water
- ddPCR Droplet Reader Oil (Bio-Rad, Catalog #:1863004)

**Consumables**

- Bio-Rad ddPCR™ 96-Well Plates (Bio-Rad, Catalog #:12001925)
- DG8™ Cartridges for QX200™/QX100™ Droplet Generator (Bio-Rad, Catalog #: 1864008)
- DG8™ Gaskets for QX200™/QX100™ Droplet Generator (Bio-Rad, Catalog #: 1864009)
- PCR PX1 Plate Sealer, foil, pierceable (Bio-Rad, Catalog #:1814040)
- Sterilized, filter tips, 200 µL, 50 µL,20 µL
- Eppendorf Tubes, 1.5mL, 2mL

**Methods**

*Primers and probe design*

The assay is designed to amplify a SARS-CoV-2 genomic RNA and sgRNA. In detail, one home-made and two previously tested assays [1] targeting 3 different regions of RNA-dependent RNA-polymerase (RdRp) of SARS-CoV-2 were used to quantify SARS-CoV-2 genomic RNA. The assay targeting the RNAseP housekeeping gene was used as reference [2]. The sgRNAs were quantified using assays adapted for the ddPCR system and targeting the envelope and nucleocapsid transcripts [3, 4]. The sequences of forward and reverse primers and of the probe are reported in S1 Table.

**Workflow of the ddPCR procedure**

SARS-CoV-2 genomic RNA and sgRNA quantification by ddPCR workflow is composed by 5 steps:

1) RNA extraction 2) master-mix preparation and sample inoculum, 3) droplets generation, 4) amplification, 5) droplets reading and data analysis.

*1. RNA extraction*

Total RNA was extracted from 280 µl of nasopharyngeal swabs using QIAamp viral RNA mini kit (Qiagen) following manufacturer’s instruction. The extracted RNA was eluted in a volume of 50 μL and stored at -80°.

*2. Master-mix preparation and sample inoculum*

The second step in the ddPCR process is the preparation of Master-mix for the subsequent ddPCR reactions. The total amount of Master-mix to be prepared depends on the total number of ddPCR reactions that should be run. For each ddPCR reaction well, a volume of 15 μL is required.

To ensure 15 μL of Master-mix for each reaction well, it is recommended to increase the volume of each reagent by 10% during preparation (total volume= 17 μL).

The specific volume for each individual reagent per reaction are reported in the table below.

Table 1. Preparation of the reaction mix.

| Reagent | Volume per reaction,  μl for 1 sample |
| --- | --- |
| Supermix | 6 |
| Primer/probe – FAM | 1 |
| Primer/probe – HEX | 1 |
| 300 mM DTT | 1 |
| Reverse Transcriptase | 2 |
| RNAse free water | 6 |
| RNA | 5 |
| Total volume | 22 |

*3. Droplets generation*

Once all the components of the master mix are assembled and the sample is added, the droplets generation step is carried out.

This step requires the use of DG8™ cartridges for the QX200™/QX100™ droplet generator (Bio-Rad, USA), which are fixed in an ad hoc DG8 cartridge holder (Bio-Rad, USA).

20μL of each reaction mixture is inoculated into the middle row of wells and 70μL of Droplet Generation Oil for Probes (Bio-Rad, USA) into the lower wells of the cartridge.

Then, each cartridge is covered with an ad hoc gasket provided by the manufacturer (Bio-Rad, USA) and placed in the QX200™ Droplet Generator instrument (Bio-Rad, USA), which can automatically generate droplets in the top row of wells.

*4. Amplification*

After droplet generation, the reaction-mix containing droplets carefully transfer droplets into a clean 96-well plate. Seal the plate using Aluminium Pierceable Foil Heat Seals with the PX1 PCR Plate Sealer.

Proceed to amplification (Table 2).

Table 2. Thermal cycling conditions.

| Cycling Step | Temperature, ℃ | Time | Ramp Rate | Number of Cycles |
| --- | --- | --- | --- | --- |
| Retro-trascriptase | 45 | 60min | 2℃/sec | 1 |
| Enzyme activation | 95 | 10min |  | 1 |
| Denaturation | 95 | 30sec |  | 40 |
| Annealing/extension | 58 | 60sec |  |  |
| Enzyme deactivation | 98 | 10min |  | 1 |
| Hold | 4 | infinity |  |  |

*5. Droplets reading and data analysis*

For the results acquisition, the plate is placed into the QX200 Droplet Reader (cat.1864003, Bio-Rad, USA). Before droplets reading, open QuantaSoft software (Bio-Rad) to set up a new plate layout and set up the reading parameters, according to the type of probes and master-mix used.

Once the raw results are obtained, it is crucial:

1. to check the total number of droplets for each well (cut-off >10,000 droplets)

2. to set a threshold line to discriminate positive and negative droplets. For this scope, the inclusion of negative and positive control is mandatory for each run of quantification in order to define the threshold.

A positive well was defined if more than 3 fluorescent signal events were shown above the threshold line.

After the definition of positivity threshold, the software generates a result expressed as copies/μL of reaction (cp/μLRx). Then, the results of each ddPCR reaction are converted the copies/mL.

**Reference**

1. WHO. Real-Time RT-PCR Assays for the Detection of SARS-CoV-2 Available at: https://www.who.int/docs/default-source/coronaviruse/real-time-rt-pcr-assays-for-the-detection-of-sars-cov-2-institut-pasteur-paris.pdf?sfvrsn=3662fcb6_220.
2. CDC. CDC’s Influenza SARS-CoV-2 Multiplex Assay. <https://www.cdc.gov/coronavirus/2019-ncov/lab/multiplex.html?CDC_AA_refVal=https%3A%2F%2Fwww.cdc.gov%2Fcoronavirus%2F2019-ncov%2Flab%2Frt-pcr-panel-primer-probes.html>.
3. Wölfel R, Corman VM, Guggemos W, et al. Virological assessment of hospitalized patients with COVID-2019. Nature 2020 581(7809):465-469.
4. Telwatte S, Martin HA, Marczak R, et al. Novel RT-ddPCR assays for measuring the levels of subgenomic and genomic SARS-CoV-2 transcripts. Methods 2022 201:15-25
