# Supplementary material for: Quantitative SARS-CoV-2 subgenomic RNA as a surrogate marker for viral infectivity: Comparison between culture isolation and direct sgRNA quantification
Source: PLoS One. 2023 Sep 1;18(9):e0291120. doi: 10.1371/journal.pone.0291120 (PMC10473502; doi:10.1371/journal.pone.0291120)
Supplement: Attachment — Submitted filename: EditorComment_Response_v.3.1.docx [file pone.0291120.s009.docx]

**PONE-D-22-24987R1**

**Title: Quantitative SARS-CoV-2 subgenomic RNA as a surrogate marker for viral infectivity: comparison between culture isolation and direct sgRNA quantification**

**Journal: PLOS ONE**

We would like to thank academic editor for his time and for the constructive criticisms he arose.

The revisions of the manuscript in accordance with his comment are summarized in our responses below.

**Response to Editor’ Comment**

1. We recommend that you deposit your laboratory protocols in protocols.io to enhance the reproducibility of your results. Protocols.io assigns your protocol its own identifier (DOI) so that it can be cited independently in the future. For instructions see: https://journals.plos.org/plosone/s/submission-guidelines#loc-laboratory-protocols. Additionally, PLOS ONE offers an option for publishing peer-reviewed Lab Protocol articles, which describe protocols hosted on protocols.io. Read more information on sharing protocols at https://plos.org/protocols?utm_medium=editorialemail&utm_source=authorletters&utm_campaign=protocols.

**Answer:** According with the editor’s comment, we provided the full protocol describing the detailed methodology used for the detection and quantification of SARS-CoV-2 subgenomic RNAs and viral load in the revised version of the manuscript as Supplementary File 1 (Page 4, Lines 114-115). We tried to deposit the protocol in protocols.io as the editor suggested, but we did not received the customer code required for the submission and requested to [plosone@plos.org](mailto:plosone@plos.org) by email in June, 2023, following the lab protocol guidelines (available at <https://journals.plos.org/plosone/s/submission-guidelines#loc-lab-protocols>). We also added a funding and data availability statement to the revised manuscript (Page 11, lines 323-329).
